# Antiviral properties and molecular docking studies of eco-friendly biosynthesized copper oxide nanoparticles against alfalfa mosaic virus

**DOI:** 10.1186/s12870-024-05802-1

**Published:** 2024-11-18

**Authors:** Dalia G. Aseel, Mona Rabie, Ali El-Far, Ahmed Abdelkhalek

**Affiliations:** 1 Plant Protection and Biomolecular Diagnosis Department, Arid Lands Cultivation Research Institute, City of Scientific Research and Technological Applications, Alexandria, 21934 Egypt; 2 Department of Botany and Microbiology, Faculty of Science, Alexandria University, Alexandria, 21511 Egypt; 3 Department of Biochemistry, Faculty of Veterinary Medicine, Damanhour University, Damanhour, 22511 Egypt

**Keywords:** *Haloxylon Salicornicum*, Copper oxide nanoparticles, Alfalfa mosaic virus, Antioxidant enzyme, Gene expression, Molecular docking

## Abstract

**Background:**

Nanotechnology has been recognized as a viable technology for enhancing agriculture, particularly in the plant pathogen management area. Alfalfa mosaic virus (AMV) is a global pathogen that affects many plant species, especially economically valuable crops. Currently, there is less data on the interaction of nanoparticles with phytopathogens, particularly viruses. The current study looked into how copper oxide nanoparticles (CuO-NPs)-mediated *Haloxylon salicornicum* aqueous extract can fight AMV infections on tobacco plants.

**Results:**

Scanning electron microscopy (SEM) and transmission electron microscopy (TEM) analyses showed that CuO-NPs have a spherical and hexagonal structure ranging from 20 to 70 nm in size. Fourier transform infrared spectroscopy (FTIR) analysis showed that the produced CuO-NPs have many functional groups and a lot of secondary plant metabolites. Under greenhouse conditions, the foliar application of CuO-NPs (100 ppm) enhanced tobacco growth and decreased viral symptoms. Treatment with CuO-NPs 48 h before (protective treatment) or 48 h after (curative treatment) AMV infection significantly reduced AMV accumulation levels by 97%. Additionally, the levels of total chlorophyll, phenolic, and flavonoid contents, as well as DPPH, exhibited a significant increase in tobacco leaves 30 days after inoculation in comparison to untreated plants. Moreover, considerable differences in levels of different antioxidant enzymes, including SOD, PPO, POX, and CAT, were also observed. On the other hand, the oxidative stress markers (MDA and H_2_O_2_) were significantly reduced in CuO-NPs-treated plants compared with non-treated plants. It was also found that the protective treatment increased the expression levels of genes involved in the jasmonic pathway (*JERF3* and *WRKY1*). On the other hand, the curative treatment increased the expression levels of polyphenolic pathway acid (*CHI* and *HQT*) and the SA-signaling pathway genes (*PR-2* and *POD*). The study of molecular docking interactions with four AMV target proteins showed that CuO-NPs had high binding energy with the viral replication protein 1a, measured at -3.2 kcal/mol. The binding with these proteins can suppress AMV replication and spread, potentially clarifying the mechanism behind the antiviral effect.

**Conclusions:**

The overall analysis results indicate that the curative treatment is more influential and successful than the protective treatment in combating AMV infection. Consequentially, CuO-NPs could potentially be employed in foliar sprays for the effective and environmentally friendly management of plant virus infections.

## Background

Nanotechnology has been recognized as a potential technology for utilization in the food sector, advancement of agriculture, and elevation of the quality of life for impoverished individuals. Moreover, it possesses several applications across every phase of the production, processing, storage, packaging, and transportation of agricultural commodities. Meanwhile, nanotechnology has emerged as a versatile technology that excels at solving problems and bridging gaps in agricultural sciences and related disciplines. Recent scientific evidence suggests that nanotechnology has a beneficial impact by mitigating agricultural issues that have adverse effects on the environment and human health while simultaneously enhancing food safety and productivity [1]. Currently, nanoparticles facilitate the precise detection and identification of plant viruses via nanobiosensor approaches [2]. Nanoparticles have demonstrated efficacy in reducing the infectivity of various phytopathogenic viruses [3]. Nonmetals, metalloids, metallic oxides, and carbon nanomaterials represent a category of nanoparticles that can effectively inhibit viral diseases in plants [2, 4].

Copper oxide (CuO), which belongs to the transition metal oxide group, has a monoclinic structure and numerous fascinating properties. It is composed of two primary elements: copper and oxygen, which correspond to the d and p blocks, respectively, and has a band gap of 2.0 eV. Copper oxide nanoparticles (CuO-NPs) are more selective and active than bulk equivalents because they have a higher surface-to-volume ratio. CuO-NPs appear as brownish-black powders [5]. CuO-NPs are one of the most widely used engineered nanoparticles, which has shown promise for usage in agriculture [6, 7]. CuO-NPs have distinctive crystal formations and feature large surface areas, rendering them extremely beneficial as antibacterial agents. Recently, Cu-based nanomaterials have been investigated as potential nanofertilizers or nanopesticides to enhance crop production, improve edible tissue nutrition, and prevent diseases, including fungal, viral, bacterial, and nematoid [8–12]. There are several techniques for CuO-NPs production, including chemical precipitation, microwave irradiation, and thermal breakdown. Nonetheless, the utilization of harmful chemicals in the chemical process limits its applicability. This has increased researchers’ interest in synthesizing CuO-NPs by biological methods, recognized for their environmental safety, ease of use, and lack of necessity for cell culture [13]. The utilization of plants for the synthesis of nanoparticles has gained popularity as a cost-effective and environmentally friendly substitute for chemical and physical methods.

Copper oxide nanoparticles can benefit plants by increasing the absorption of vital nutrients like magnesium, iron, phosphates, and others. While CuO-NPs are generally regarded as harmless when used as nutrition, multiple studies have demonstrated their toxicity due to the generation of oxidative stress at greater concentrations [14]. So, it is very important to study how harmful nanoparticles are to plant tissues by looking at how reactive oxygen species (ROS) form in cells after a non-lethal dose is applied. The influence of CuO-NPs on plants is dependent on the species, concentration, and method of application. The effects of CuO-NPs on plants can vary depending on the concentration and duration of exposure. Monocots have a greater susceptibility to CuO-NPs, while dicots display varied reactions [15]. The release of Cu^2+^ ions may lead to the creation of several ROS molecules, including hydrogen peroxide, malonaldehyde, and hydroxyl radicals, resulting in damage to the tissue cell membrane [16]. At a concentration of 1000 mg/L, CuO-NPs adversely affect photosynthetic indices, transpiration rates, and stomatal conductance while also inhibiting the germination rate of *Oryza sativa* [17]. In many cases, optimal dosage selection is critical for agricultural applications of CuO-NPs. Concentrations of CuO-NPs less than 100 µg/L had a positive or neutral effect on root and shoot growth, whereas higher concentrations inhibited growth. The reaction to CuO-NPs concentration suggests that there may be an optimal concentration range for promoting plant development. However, above this range, the harmful effects become more apparent [18].

Alfalfa mosaic virus (AMV) is a plant virus classified under the family *Bromoviridae* and the genus *Alfamovirus*. AMV is a ubiquitous virus that has a significant impact on numerous plant species, particularly economically valuable crops [19]. It has a tripartite genome composed of single-stranded positive-sense RNAs, of which RNA1 and RNA2 encode the viral replicase proteins P1 and P2, respectively, and RNA3 encodes the movement protein (MP) and coat protein (CP) [20]. The CP is translated from a subgenomic messenger RNA4, which is synthesized during the replication of RNA3 [21]. The symptoms of AMV encompass mosaic patterns, mottled appearances, deformities characterized by calico blotching, and necrosis [22]. The transmission of the virus can occur through aphids in a non-persistent manner, as well as through mechanical means and plant seeds [23, 24]. In addition, both weeds and cultivated plants have a significant impact on the spread of AMV since they serve as reservoirs for the virus and provide a habitat for aphids during the summer, hence enhancing the longevity and prevalence of AMV [23, 25]. The jasmonic biosynthetic pathway (*JERF3* and *WRKY1*) has a role in plant immunity [26]. Plant polyphenolic compounds, also called secondary metabolites, are known to help plants grow and protect them against biotic and abiotic stresses [27]. The salicylic acid (SA)-signaling pathway, where reactive oxygen species (ROS) stimulate programmed cell death at plant cell infection sites, plays an important role as a signaling molecule for the activation of antioxidant activity and the induction of the genes that code for pathogenesis-related proteins (PRs) [28].

Plant extracts from *Malva sylvestris* [29], *Ocimum basilicum* [30], *Punica granatum* [31], and *Ficus sycomorus* [32] have been reported as reducing agents to biosynthesized nanoparticles. Multiple plant species have been investigated for the production of CuO-NPs [33]. Plant-based CuO-NPs have also been used successfully in several biological and non-biological tasks, such as cytotoxicity [34], antibacterial activity [33], and photocatalytic activity [35]. *Haloxylon salicornicum* belongs to the *Amaranthaceae* family and is classified as a shrub or subshrub. It is distributed across various global locations, including Egypt, Pakistan, Bahrain, Iraq, Palestine, Jordan, and the Kingdom of Saudi Arabia. According to Ullah et al. [36], it has multiple uses, including its effectiveness as an antiseptic, anti-diabetic, and anti-inflammatory agent. It was reported that the aerial parts are abundant in bioactive compounds, such as alkaloids, piperidine, and terpenes. These compounds exhibit a variety of biological activities, including antituberculosis properties, hepatoprotective effects, and functions as human antimicrobial agents [37]. However, there are currently no research articles that clearly explain the impact of *H. salicornicum* extracts on plant diseases or their usage as stabilizing agents in nanoparticle production biosynthesis. Consequently, the present study aimed to utilize the aqueous extract of *H. salicornicum* as a stabilizing agent to achieve the eco-friendly production of CuO-NPs. Several analysis techniques were used to characterize the biosynthesized CuO-NPs. Furthermore, we evaluated the effects of synthesized CuO-NPs on viral symptoms, plant development, and AMV accumulation in infected tissues. Several physio-biochemical analyses were assessed. In addition, the effectiveness of CuO-NPs in inducing systemic acquired resistance against AMV and its effects on the transcriptional levels of several-defense related genes were also studied. Additionally, the molecular docking between CuO-NPs and the AMV target proteins was studied. These proteins include the capsid protein, the movement protein, RNA-directed RNA polymerase 2a, and replication protein 1a. This study offers a promising strategy for plant protection, using CuO-NPs as elicitor molecules to trigger systemic acquired resistance (SAR) and control plant viral disease.

## Materials and methods

### Plant material and source of viral isolate

The *H. salicornicum* L. plants were obtained from an open field (30.892253, 29.546623) located in New Borg El-Arab city, Alexandria Governorate, Egypt. *H. salicornicum* was identified by Prof. Dr. El-Sayed F. El-Halwany, Professor of Plant Ecology, Botany Department, Faculty of Science, Mansoura University, Egypt. The virus-free seeds of *Nicotiana glutinosa* L., a type of tobacco, were obtained from the Agriculture Research Center, Ministry of Agriculture, Egypt. The AMV strain KH1 (Accession number MN099289) utilized in this investigation was previously assessed [38] and consistently cultivated on potato plants under a controlled greenhouse condition.

### Preparation of plant extract and synthesis of CuO-NPs

The CuO-NPs were synthesized using *H. salicornicum* extract as a mediator and cupric sulfate (CuSO_4_, Sigma-Aldrich, USA) as a precursor, following the procedure outlined by Chen et al. [39] with minor modifications. In summary, the fine plant leaf powder was thoroughly mixed in double-distilled water with a final ratio of 10% solid material to liquid in a shaking water bath (Thermo Fisher Scientific Inc., Waltham, MA, USA) at a speed of 150 rpm for 3 h at 80 ^o^C. Subsequently, the plant extract supernatant was obtained by centrifugation at 10,000 rpm for 15 min. The filtrate was then used as a reducing agent. A 10 mM CuSO_4_ solution was mixed directly with a filtered plant extract in a 1:1 ratio and agitated at 50 °C for 5 h. The appearance of a dark brownish-to-black color in the reduction mixer indicated that CuO-NPs were developing. CuO-NPs were separated by centrifugation at 10,000 rpm for 15 min. To assure purity, the precipitated CuO-NPs were washed with 95% ethanol and then rinsed many times with double-distilled water to eliminate any remaining ethanol. They were then dried for 24 h at 80 °C before any other procedures were carried out.

### Characterization of CuO-NPs

The green synthesized CuO-NPs were analyzed using various instrumental techniques. The structural morphology, shape, and size of the synthesized particles was examined using scanning electron microscopy (SEM) with an acceleration voltage of 15 kV and magnifications of 20,000_X_. The JSM-6360 LA microscope from Tokyo, Japan was used for this purpose. Transmission electron microscopy (TEM) is a very good electron microscopy method used to study CuO-NPs and other metal nanoparticles and figure out their shape. In the TEM technique, an intense beam is transmitted through a very thin sample and interfaces, resulting in the formation of a picture. A copper grid coated with carbon was used to create thin films of the produced nanoparticles. Approximately 20 µL of an aqueous suspension of CuO-NPs was applied to the grid. The slide was cleansed using 15 droplets of distilled water, stained with 1% uranyl acetate for optimal contrast, placed onto grids, and inspected after a duration of one minute. Subsequently, the image was evaluated while operating at a voltage of 120 kV. At 25 ^o^C, Zetasizer Nano Z 590 (Malvern, Granta Lodge, UK) equipment was used to assess the particle size distribution of biosynthesized CuO-NPs via dynamic light scattering (DLS). Fourier transform infrared (FTIR) spectroscopy (FTIR-8400 S, Shimadzu, Tokyo, Japan) was utilized to examine the various functional groups associated with the synthesized nanoparticles. The FTIR conducted with the KBr-disc method, covering a range of 400–4000 cm^–1^.

### Greenhouse experimental design and assessment of plant growth parameters

The effects of CuO-NPs on tobacco plants were assessed under controlled greenhouse conditions. The study consists of five different treatments. Every treatment consisted of five replicates or pots. Every pot held five tobacco plants. Each pot contained soil that underwent sterilization and comprised an equal blend of clay and sand in a 1:1 ratio. The initial treatment (control) involved the healthy plant, which was inoculated with a virus-free inoculation buffer. The second treatment, known as AMV, is specifically intended for those who are only afflicted with the virus. The third treatment was alone, treated with a concentration of 100 ppm of CuO-NPs. The fourth treatment, which was exposed to AMV 48 h prior to treatment with CuO-NPs at 100 ppm, had a protective effect against the virus. The fifth treatment, which involved the application of CuO-NPs at 100 ppm followed by AMV infection after 48 h, was deemed to have a curative effect on the plant prior to inoculation. Using the forefinger method, carborundum was applied to two true upper leaves, and they were then put in contact with a freshly prepared AMV inoculum [40]. The pots were then grown in a controlled greenhouse with 27 ± 2 ºC, 70% relative humidity, 16 h of light, and 8 h of darkness. The pots were examined daily for a period of 28 days to document the development of symptoms. Additionally, the impact of CuO-NPs on various growth characteristics was assessed, including the fresh and dry weights of both shoot and root (g), the lengths of shoot and root (cm), and the number of leaves.

### Determination of total chlorophyll and DPPH contents

A 500 mg of tobacco leaves were ground in a mortar with 5 mL of 80% acetone, incubated for a day at 4 ^o^C, centrifuged at 4000 rpm for 25 min, and then measured at 645 and 663 nm following these equations:

$${\rm{Chl}}\,{\rm{a}}\,({\rm{mg}}/{\rm{mL}})\, = \,(11.64\, \times \,{\rm{A}}\,663)\, - \,(2.16\;\, \times \;{\rm{A}}\,645)$$

$${\rm{Chl}}\;{\rm{b}}\;({\rm{mg}}/{\rm{mL}})\; = \;(20.97\; \times \;{\rm{A}}\;645)\; - \;(3.94\; \times \;{\rm{A}}\;663)$$

For DPPH, 500 mg of the leaves powder was combined with 5 mL of methanol (96%), and this solution was put in a shaker at 80 rpm for 48 h at 25 ^o^C according to the method of Moattar et al. [41]. The sample was filtered using Whatman No. 3 filter paper, and then the reaction started by adding 0.5 mL of the extracted plant to 3.5 mL of freshly prepared DPPH (2,2-diphenyl-1-picryl-hydraziy) methanol solution into a tube. After 30 min of incubation at room temperature in the dark, followed by reading the absorbance at 517 nm.

### Determination of phenolic and flavonoid content

We used the Hamrouni-Sellami et al. [42] method to measure the phenolic content. We added a 0.125-mL portion of the diluted methanol sample to a mixture consisting of 0.125 mL of Folin-Ciocalteu reagent and 0.5 mL of deionized water. After shaking the mixture, we let it sit for 6 min before adding 1.25 mL of 7% Na_2_CO_3_. Ultimately, add deionized water to the solution until it reaches a total volume of 3 mL. Proceed to vigorously mix the solution, and then allow it to incubate for a duration of 90 min at a temperature of 23 °C. Measure the optical density at a wavelength of 760 nm. Gallic acid was employed as a standard curve within the concentration range of 50–400 mg/mL.

Regarding the concentration of flavonoids, Zafar et al. [43] method using aluminum chloride were used. The approach involves adding 20 µl of a sample to a mixture of 10 µL of 10% aluminum chloride and 10 µL of 1 M potassium acetate. Subsequently, introduce distilled water until the total volume reaches 200 µL. Subsequently, allow the mixture to incubate for a duration of 30 min at ambient temperature, followed by measuring the absorbance at a wavelength of 415 nm.

### Determination of antioxidant enzymes activity

The activity of superoxide dismutase (SOD) was determined by conducting a reaction using 200 µL enzyme extract, 200 µ pyrogallol solution (7.2 mM in H_2_O), 3 mL of 50 mM Tris HCL (pH = 8.5), and 10 mM EDTA. After 10 min, the reaction was stopped by adding 1 ml of 1 N HCL [44]. The percentage of scavenging activity can be calculated using the equation: Scavenging activity % = 100 - [(A - B) / C] * 100, where A represents the absorbance reading at 420 nm. Employing ascorbic acid as a positive control within the concentration range of 0.125 to 2 mg/mL. A represents the absorbance of the sample or standard when the reagent is present, B represents the absorbance of the sample or standard when the reagent is absent, and C represents the absorbance of the control.

To measure the activity of polyphenol oxidase (PPO) as described by Lee et al. [45], the reaction was initiated by combining 100 µL of 100 mM pH = 7 sodium phosphate buffer, 50 µL of 150 mM catechol, and 50 µL of enzyme extract. Measure the amount of light absorbed at a wavelength of 404 nm. The peroxidase activity (POX) was measured by combining 125 µL of sodium phosphate buffer (100 mM, pH = 7), 25 µL of guaiacol (24 mM), 25 µL of enzyme extract, and finally 25 µL of H_2_O_2_ (12 mM). Then, the absorbance at 465 nm served to measure the activity [45]. In the case of catalase (CAT), the reaction was quantified by mixing 750 µL of phosphate buffer with a pH of 7, 30 µL of enzyme extract, and finally 30 µL of 15 mM H_2_O_2_. The absorbance at 240 nm was then measured, following the procedure described by Pan and Zhang [46], as per the given equation. CAT (mM/g FW) = (activity A V/a) = (E × W). The activity value is equal to the optical density (OD) value, the sample weight is W, the buffer solution used to extract the enzyme is V, the amount of enzyme extract used in the test solution is a, and the exclusion coefficient is 39.4 mM/cm.

### Determination of oxidative stress markers

To measure the concentration of malonaldehyde (MDA), 0.1 g of tissue were taken and grinding it in 1.5 ml of TCA (thiobarbituric acid). Transfer the resulting mixture into an Eppendorf tube and subject it to centrifugation at a speed of 12,000 rpm for 15 min [47]. Next, add 0.5 mL of the resulting supernatant to 1 mL of TBA and TCA. Heat the mixture in a water bath at a temperature of 95 °C for a period of 20 min, and promptly transfer it to an ice bath afterwards. Perform centrifugation on the sample for a duration of 10 min, then measure the absorbance at wavelengths of 532 and 600 nm using the provided formula. The concentration of TBARS (nmol/g FW) can be calculated using the formula:

$$[({\rm{OD}}532 - {\rm{OD}}600)\; \times \;{\rm{A}}\; \times \;{\rm{V}}] \div ({\rm{a}}\; \times \;{\rm{E}}\; \times \;{\rm{W}})$$

The variables in the equation are defined as follows: A represents the entire assay volume, V represents the total volume of phosphate buffer used for enzyme extraction, a represents the volume of supernatant used, W represents the fresh weight of the sample, and E represents the extinction coefficient (1.55 × 102). The value is 155 millimeters per centimeter.

Regarding hydrogen peroxide (H_2_O_2_): Mix 0.2 g of freshly picked leaves with 2 mL of pre-chilled 0.1% TCA (tri-chloroacetic acid), make sure the leaves are well mixed, and then spin the mixture at 12,000 rpm for 15 min. Follow the steps in Velikova et al. [48] and mix 50 µL of the supernatant with 50 µL of a 10 mM potassium phosphate buffer at pH 7 and 100 µL of potassium iodide. Measure the amount of light absorbed at a wavelength of 390 nm.

### Impact of CuO-NPs on AMV accumulation and defense-related genes

The TRIZOL reagent kit (Life Technologies, Invitrogen, CA, USA) was employed to extract total RNA from 100 mg of fresh plant tissue, adhering to the manufacturer’s guidelines. The extracted RNA was dissolved in DEPC-treated water and quantified using a UV spectrophotometer (DU730, Beckman Coulter Inc., Brea, CA, USA). To convert the extracted RNA into cDNA, we used a reverse transcription procedure, employing M-MuLV reverse transcriptase from Biolabs, New England, inside a thermal cycler. Utilizing 1 µg of RNA treated with DNase I from each sample as a template to generate cDNA [49]. Subsequently, the cDNA was stored at -20 °C for future utilization in RT-qPCR experiments. The RT-qPCR was performed using the SYBR^®^ green kit (Bioloine, Luckenwalde, Germany), as reported by Aseel et al. [50]. To sum up, the RT-qPCR was made up of 10 µL of SYBR^®^ green master mix, 1 µL of forward primer (10 pM), 1 µL of reverse primer (10 pM), and 7 µL of Milli-Q water that had been sterilized twice. The reactions were run in a Rotor-Gene 6000 thermal cycler as previously described [50]. Table 1 illustrates the list of primers used that targeted the *AMV-CP* gene and many defensive genes, including *JERF3* and *WRKY1* from the Jasmonic pathway genes, *CHI* and *HQT* from the polyphenolic pathway genes, and *PR-2* and *POD* from pathogenesis-related protein-encoding genes. The expression of *AMV-CP* gene was utilized to assess the degree of viral accumulation within plant tissues. The housekeeping gene *EF1-a* was utilized to normalized the expression of the genes being investigated. The relative expression of these genes was determined using the 2^−∆∆CT^ method [51].

**Table 1:** Primer sequences used in RT-qPCR for defense expression genes in this study

Gene	Abbreviation	Primer Sequence (5′–3′)	Related Pathway
WRKY transcription factor 1	*SbWRKY1-F*	CGTGCAGCAGCAAAGCAA	ET/JA-signaling pathways
	*SbWRKY1-R*	GTCGCAGGTATGCTCGTTGA	
Jasmonate and ethylene-response factor 3	*JERF3-F*	GCCATTTGCCTTCTCTGCTTC	
	*JERF3-R*	GCAGCAGCATCCTTG TCT GA	
Peroxidase	*POD-F*	CCTTGTTGGTGGGCACACAA	SA-signaling pathway
	*POD-R*	GGCCACCAGTGGAGTTGAAA	
β-1,3-glucanases	*PR2-F*	TATAGCCGTTGGAAACGAAG	
	*PR2-R*	CAACTTGCCATCACATTCTG	
Hydroxycinnamoyl CoA quinate hydroxycinnamoyl transferase	*HQT-F*	CCCAATGGCTGGAAGATTAGCTA	Polyphenol synthesis pathway
	*HQT-R*	CATGAATCACTTTCAGCCTCAACAA	
Chalcone isomerase	*CHI-F*	GGCAGGCCATTGAAAAGTTCC	
	*CHI-R*	CTAATCGTCAATGATCCAAGCGG	
Elongation factor 1-α	*EF1-a-F*	GAACTGGGTGCTTGATAGGC	Housekeeping
	*EF1-a-R*	AACCAAAATATCCGGAGTAAAAGA	
Alfalfa mosaic virus-Capsid Protein	*AMV-CP-F*	ATGCCATTCTCCGTCTTGACTTG	Virus assembly
	*AMV- CP-R*	GAGTTGTATGTAGTCTCGTGGATT	

### Evaluation of the interaction between CuO-NPs and AMV genes using molecular docking

The UniProt database (https://www.uniprot.org/) was used to get the protein sequences of the AMV’s capsid protein, movement protein, RNA-directed RNA polymerase, and replication protein 1a. The three-dimensional structures of these proteins were generated using the Robetta server (https://robetta.bakerlab.org/) (Beak et al., 2021). Additionally, the three-dimensional configuration of CuO was acquired from the PubChem database (https://pubchem.ncbi.nlm.nih.gov/). The target proteins and CuO structures were converted to mol structure format using UCSF Chimera 1.16 (University of California, San Francisco, CA, USA) in preparation for docking. In addition, molecular docking was performed using AutoDock 4.2.6, a software developed by the Scripps Research Institute in La Jolla, CA, USA. The docking process was carried out within the UCSF Chimera software. The ligand-protein complexes that were docked together were visualized using the BIOVIA Discovery Studio 2016 Client program [52].

### Statistical analysis

The data was analyzed using ANOVA with the CoStat program. Additionally, Turkey’s honest significant differences approach (HSD) was employed to detect statistical differences based on the probability level in the mean and standard deviation displayed in the column bars.

## Results and discussion

### Analyzing the characteristics of the greenly synthesized CuO-NPs

**Fig. 1:**
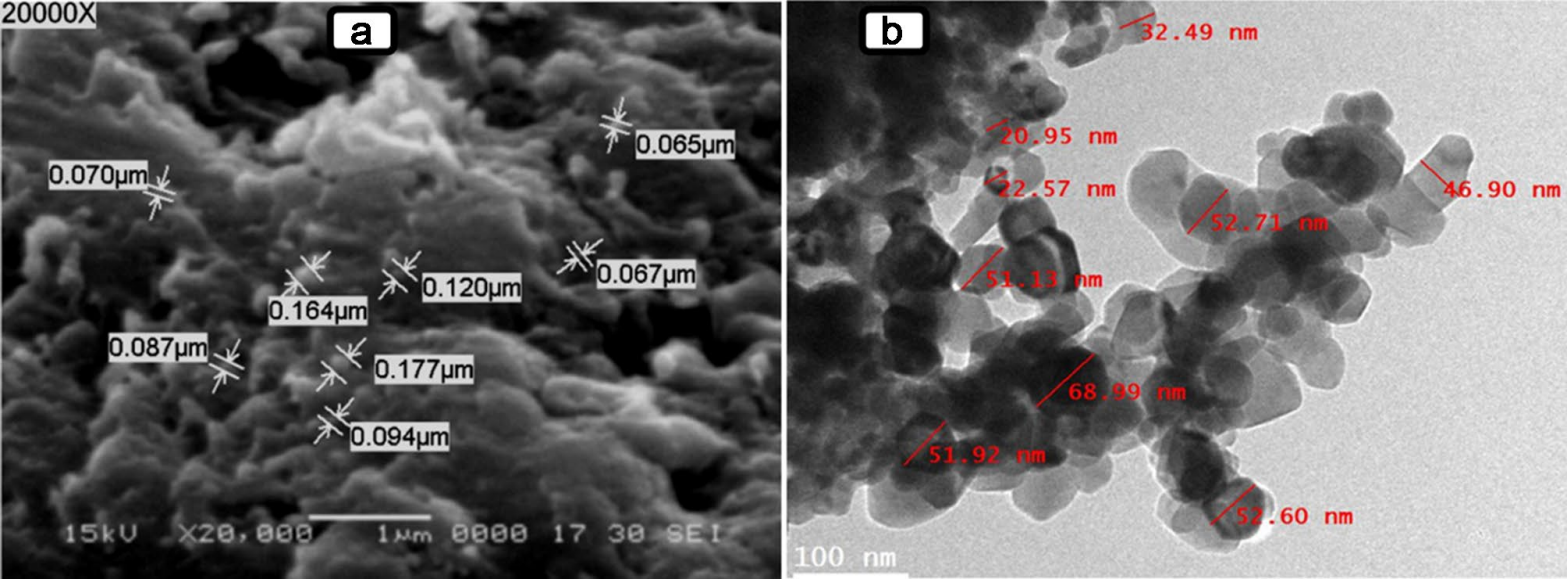
Images showed that the morphological and particle characterization of the prepared CuO-NPs synthesis using *H. salicornicum* extract, illustrated by SEM analysis at 20000x (**A**) and TEM at Scale bar of 100 nm (**B**)

**Fig. 2:**
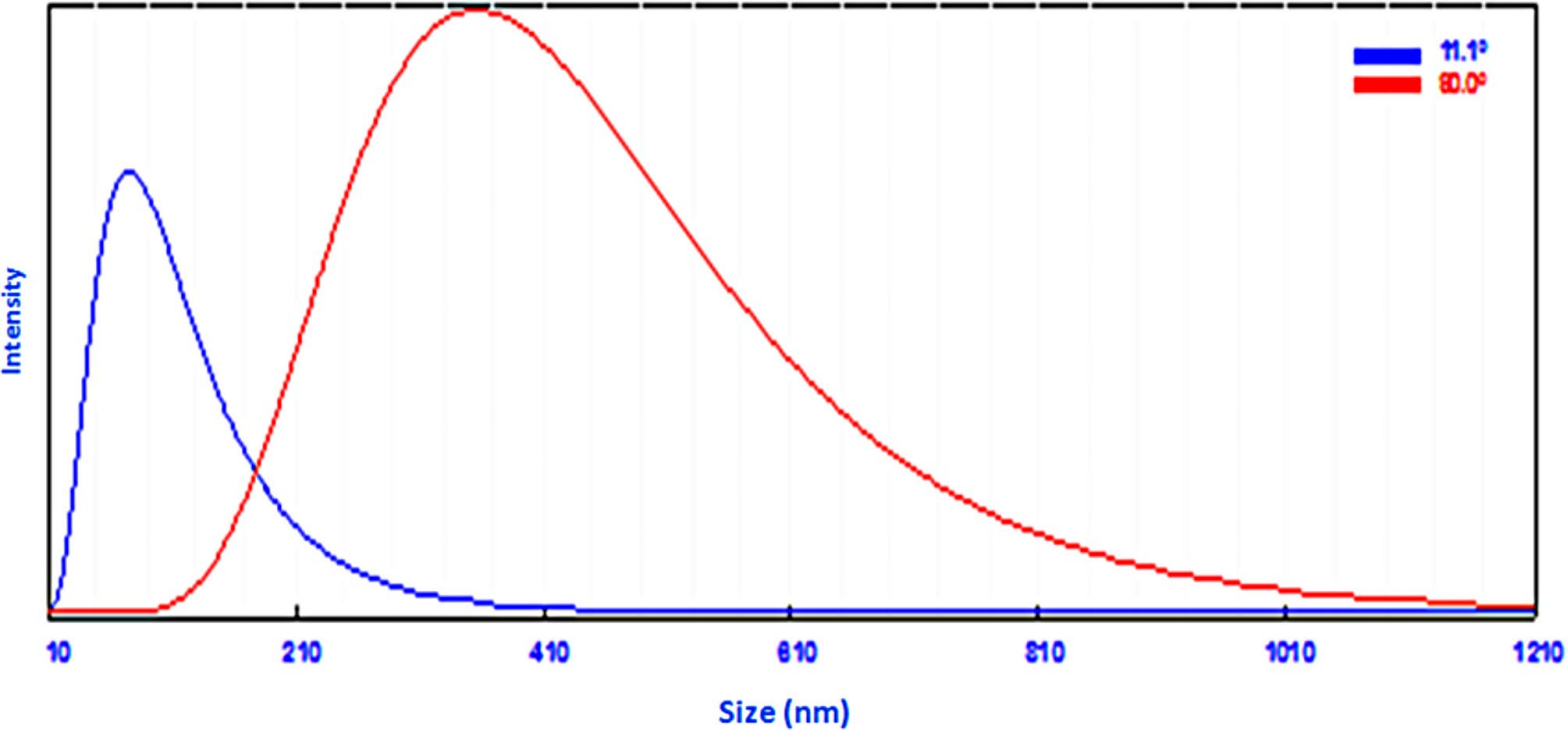
The particle size distribution of the prepared CuO-NPs synthesis using *H. salicornicum* extract through Zetasizer’s two angles 11.1 ° and 90 °

The SEM image analysis revealed CuO-NPs with a spherical morphology with varying sizes of 65 to 177 nm. This spherical shape is due to the stabilizing and capping effects of phytochemicals found in plant extracts, which stop the assembled particles from sticking together (Fig. 1A). Nanoparticles can be produced in large quantities using the applied method. This is consistent with the findings of Altikatoglu et al. [53], who found a well-dispersed, spherical-shaped distribution of CuO-NPs with *Ocimum basilicum* extract and particle sizes of less than 70 nm. TEM reveals various sizes of CuO-NPs, ranging from 20 to 70 nanometers in diameter, as shown in Fig. 1B. The CuO-NPs produced are crystalline and exhibit nearly spherical forms [53]. The particle size distribution seen in the TEM figure is consistent with the size range of CuO-NPs reported in earlier investigations [54–56]. The DLS analysis of particle size was determined using two distinct scattering angles (11.1° and 90°). At an angle of 11.1°, the average particle size was found to be 84.8 nm, with a P.I. of -9.984 and a coefficient of 5.05 e-12. At an angle of 90°, the average recorded value was 363 nm, with P.I. coefficients of 0.101 and 1.18e-12 (Fig. 2). The DLS data indicated that the nanoparticles were larger than those seen in SEM and TEM micrographs, particularly at a 90° angle. This could be attributed to the effects of Brownian motion [57].

**Fig. 3:**
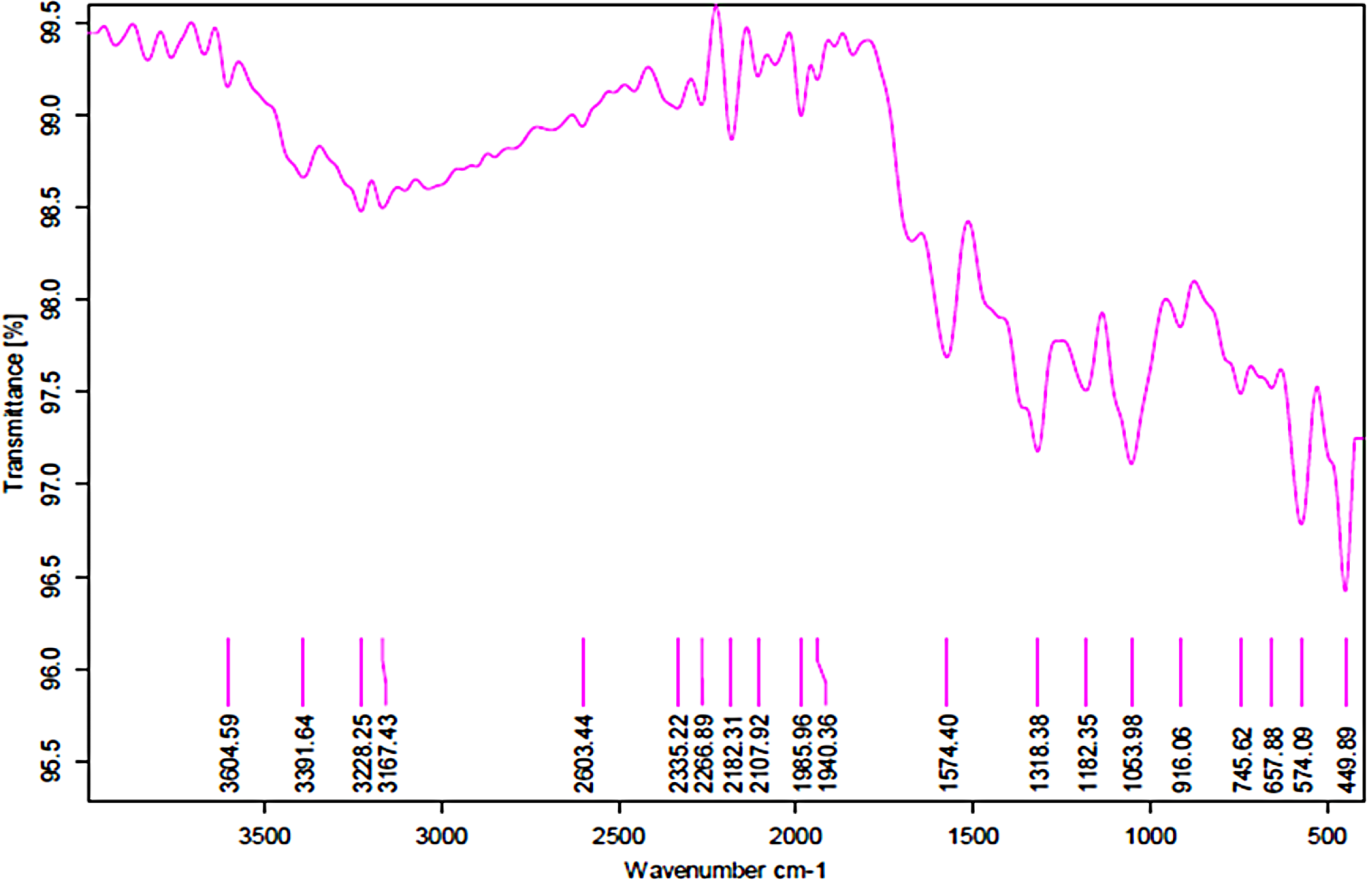
FTIR spectrum of CuO-NPs synthesis using *H. salicornicum* extract

The spectrum of FTIR showed the CuO-NPs functional groups in the range of 4000 –400 cm^− 1^ (Fig. 3). According to the infrared (IR) spectrum, there are several peaks. The peaks that were found associated with CuO-NPs correspond to O-H, C = O, C-N, C-H, and C = C. The data showed several medium-sharp bands that recognized the absorption at 3000–3604 cm^− 1^ that indicated (O-H) alcohols (phenols or N-H amines) and the peak that showed at 2603 cm^− 1^ to C-H stretching. The absorption peaks at 2000–2400 cm^− 1^ showed C = O and C = C. Peaks at 1940 cm^− 1^ and 1980 cm^− 1^ represent C-H bending of aromatic compounds. At 1574 cm^− 1^ the peak suggests N-O stretching; another peak at 1000–1400 cm^− 1^ represents O-H bending. The peaks observed between 400 and 1000 cm^− 1^ correspond to O-H bending and C-C stretching (Fig. 3). That agreement is with Bhatnagar et al. [58], where the bands found at around 3253 and 2948 cm^− 1^ indicated the bonds related to the type movements of stretching vibrations in primary and secondary amines, and the broad and strong absorption band at 2356 cm^− 1^ paralleled the C-H stretching aldehydes. While the absorption peaks at 2000–2400 cm^− 1^ showed C = O and C = C, which agree with Shahzad et al. [59]. In addition, peaks at 1940 cm^− 1^ and 1980 cm^− 1^ represent C-H bending of aromatic compounds. At 1574 cm^− 1^ the peak suggested N-O stretching; other peaks at 1000–1400 cm^− 1^ represent O-H bending; that is likely Ramasubbu et al. [60], who noted a broadband to O-H bending at 1401 cm^− 1^. In the same case, Aseel et al. [50] found a large peak at 3611.60 cm^− 1^ that might be the result of inner-surface hydroxyl group (O-H) stretching and suggests the existence of hydrogen-bonded groups. Also, these findings confirm earlier findings that the stretching vibration of OH groups in the structures of allophane and imogolite is what causes the broadband at 3432 cm^− 1^ to exist [61, 62]. According to this IR spectrum, nucleic acids, carbohydrates, proteins, alkaloids, phenols, and flavonoids serve as capping agents for the nanoparticles, which decrease and stabilize them [63]. These metabolites and compounds preserve the nanoparticles’ structure in alkaline conditions [64].

### Impact of CuO-NPs on symptoms appearance, growth parameters and viral accumulation level

CuO-NP foliar spray at 100 ppm improved all growth indices, including plant length, fresh weight, dry weight of the shoot and root system, and plant leaf number (Table 2). In terms of plant length, the control treatment had the longest shoots (7.6 ± 1.04 cm), followed by the CuO-NPs treatment with 6.3 ± 1.26 cm and the curative treatment with 6.5 ± 0.50 cm. The protective treatment had the longest roots (16.6 ± 3.21 cm). Regarding fresh weight, the CuO-NPs treatment exhibited the highest shoot and root weight with 3.83 ± 0.21 and 1.50 ± 0.14 g, respectively, with no significant change with protective treatment. In terms of dry weight, the curative treatment showed the highest shoot dry with 0.421 ± 0.03 g, while CuO-NPs showed the highest root dry with 0.323 ± 0.02 g. For plant leaf number development, the AMV treatment showed the lowest developed number of leaves with 8.3 ± 0.58, while there were different significant changes between other fur treatments (Table 2). The ZnO-NPs were shown to enhance tomato plants grow and develop against TMV [65], and Ag-NPs treatment in tomato plants lowered the disease severity and amount of ToMV or PVY in plant tissues [66].

**Table 2:** Impact of CuO NPs_100ppm_ on the growth development of tobacco plants infected with AMV (28 days after inoculation). Healthy tobacco plants (control); tobacco plants inoculated with virus only (AMV); tobacco plants treated with CuO-NP100ppm alone (CuO-NPs); tobacco plants inoculated with CuO-NPs_100ppm_ 48 h after AMV inoculation (curative treatment); tobacco plants inoculated with CuO-NPs_100ppm_ 48 h before AMV inoculation (protective treatment)

Treatments	Length ± SD		Fresh weight ± SD		Dry weight ± SD		Leaves number
	Shoot	Root	Shoot	Root	Shoot	Root	
Control	7.6 ± 1.04 a	09.5 ± 0.50 bc	2.91 ± 0.60 bc	0.97 ± 0.14 ab	0.32 ± 0.01 b	0.12 ± 0.01 c	15.0 ± 1.00 a
AMV	3.8 ± 0.29 c	05.8 ± 1.61c	2.23 ± 0.25 c	0.69 ± 0.04 b	0.15 ± 0.02 c	0.08 ± 0.01 d	8.3 ± 0.58 b
CuO-NPs	6.3 ± 1.26 ab	11.5 ± 1.00 ab	3.83 ± 0.21 a	1.50 ± 0.14 a	0.33 ± 0.02 b	0.32 ± 0.02 a	19.3 ± 3.06 a
Curative	6.5 ± 0.50 ab	13.5 ± 2.29 ab	3.36 ± 0.23 ab	1.23 ± 0.08 a	0.42 ± 0.03 a	0.18 ± 0.01 b	19.1 ± 2.08 a
Protective	5.3 ± 0.70 bc	16.6 ± 3.21 a	3.86 ± 0.23 a	1.32 ± 0.08 a	0.31 ± 0.01 b	0.19 ± 0.01 b	16.6 ± 1.53 a

Differences between groups were determined using a one-way analysis of variance (ANOVA) and Tukey’s HSD test at the *p* ≤ 0.05 significance level in the CoStat statistical package. Statistical significance was indicated alphabetically above the histogram in ascending order, whereas a > b > c > d

**Fig. 4:**
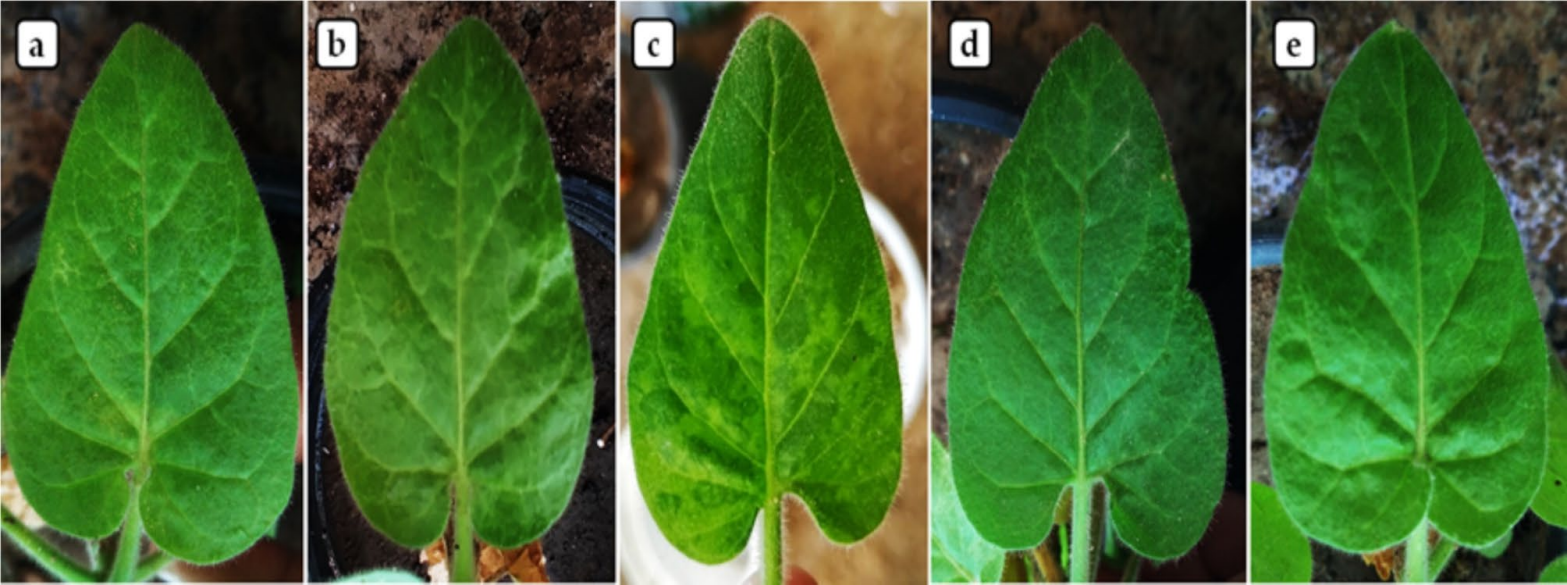
Impact of the foliar application of CuO-NPs synthesis using *H. salicornicum* extract on AMV on tobacco plants under greenhouse conditions. **a**: control tobacco plants; **b**: tobacco plants inoculated with CuO-NPs100ppm; **c**: tobacco plants inoculated with AMV; **d**: tobacco plants inoculated with CuO-NPs100ppm 48 h after AMV inoculation (curative); **e**: tobacco plants inoculated with CuO-NPs100ppm 48 h before AMV inoculation (protective)

Nanoparticles have been shown to have viricidal action against a variety of viruses and reduce viral infectivity [67]. The NPs stop the binding, fusion, replication, and infectivity of virion. Additionally, they might prevent the virus from recognizing its host plant and entering it. Via glycoprotein receptors, the NPs engage with virus surface proteins, preventing the host cells from recognizing the virus. The effectiveness of ZnO-NPs and SiO_2_-NPs against TMV was reported by Cai et al. [68]. The findings showed that the interaction of NPs with the glycoprotein envelope inhibits the direct inactivation of TMV by Me-NPs. This interaction causes direct damage to the TMV shell protein, influences aggregation. In our study, under greenhouse settings, the *N. glutinosa* plants infected with AMV showed distinct discoloration and severe mosaic symptoms compared to the control plant at 15 dpi (Fig. 4). In the protective treatment, only mild mosaic symptoms were observed (Fig. 4). No viral symptoms were observed in *N. glutinosa* plants treated with CuO-NPs alone or with a curative treatment, as well as control plants. The results align with previous studies that documented the influence of nanoparticles on viral symptom development [69, 70]. The RT-qPCR assay (Table 3) showed that CuO-NPs treated plants greatly reduced or suppressed AMV accumulation transcriptional levels in curative treatment (3.23-fold) and protective treatment (3.96-fold) compared with plants inoculated with AMV only (178.93-fold). The most important gene of the viral genome, AMV-CP, is a necessary criterion for characterizing the classification and identification of the virus [71].

**Table 3:** Impact of CuO NPs_100ppm_ on the AMV accumulation levels of tobacco plants infected with AMV (28 days after inoculation). Healthy tobacco plants (control); tobacco plants inoculated with virus only (AMV); tobacco plants treated with CuO-NP100ppm alone (CuO-NPs); tobacco plants inoculated with CuO-NPs_100ppm_ 48 h after AMV inoculation (curative treatment); tobacco plants inoculated with CuO-NPs_100ppm_ 48 h before AMV inoculation (protective treatment)

Treatments	Relative expression level ± SD	Decreased in AMV accumulation level (%)
Control	0 ± 0 c	100
AMV	178.93 ± 5.36 a	0
CuO-NPs	0 ± 0 c	100
Curative	3.23 ± 0.98 b	98.1
Protective	3.96 ± 0.87 b	97.7

Differences between groups were determined using a one-way analysis of variance (ANOVA) and Tukey’s HSD test at the *p* ≤ 0.05 significance level in the CoStat statistical package. Statistical significance was indicated alphabetically above the histogram in ascending order, whereas a > b > c > d

### Impact of CuO-NPs on chlorophyll and DPPH contents

The curative treatment exhibited the highest chlorophyll concentration, measuring 5.43 mg/g, in comparison to the control plant, which had a chlorophyll content of 4.69 mg/g. However, with the protective treatment, the chlorophyll a content reached a record high of 3.21 mg/g, while the treatment with the AMV resulted in the lowest amount of chlorophyll a content at 1.97 mg/g. Furthermore, in chlorophyll b, the curative and CuO-NP_100ppm_ treatments had the most elevated levels of content, measuring 3.72 mg/g and 3.33 mg/g, respectively. The control plant exhibited a measurement of 3.29 mg/g after being treated with protection and AMV alone, while the reported measurements for protection and AMV were 3.01 mg/g and 2.46 mg/g, respectively. Increased CuO-NPs concentrations decreased the total chlorophyll levels in soybean plants, according to a study by Nair and Chung [56]. A recent study demonstrated that altering the concentration of CuO-NPs has the potential to induce variations in the length of the roots and shoots, the vigor index, and the total chlorophyll content in soybeans. Elevating this concentration can have harmful effects and lead to reduced germination and overall chlorophyll levels [72]. DPPH is commonly regarded as a stable free radical and is primarily employed for in vitro assessment of antioxidant research [60]. The DPPH analysis revealed that the curative treatment exhibited a higher content of 16.93 mg/g compared to the protective treatment, which recorded 10.59 mg/g. On the other hand, the treatments of CuO-NP100 ppm in the control plant and AMV reported 7.13 mg/g, 5.94 mg/g, and 3.82 mg/g, respectively (Table 4). In their 2021 study, Peddi et al. discovered that CuO-NPs made it easier to stop radicals and looked into how they affected the removal of DPPH radicals in *Suaeda maritima* (L.) Dumort.

**Table 4:** Impact of CuO-NPs_100ppm_ on the non-enzymatic activities of tobacco plants infected with AMV (28 days after inoculation). Healthy tobacco plants (control); tobacco plants inoculated with virus only (AMV); tobacco plants treated with CuO-NP100ppm alone (CuO-NPs); tobacco plants inoculated with CuO-NPs_100ppm_ 48 h after AMV inoculation (curative treatment); tobacco plants inoculated with CuO-NPs_100ppm_ 48 h before AMV inoculation (protective treatment)

Treatments	Non-Enzymatic activities ± SD				
	Chlorophyll a	Chlorophyll b	Phenolic	Flavonoid	DPPH
Control	4.69 ± 0.13 b	3.29 ± 0.23 ab	397.5 ± 7.35 a	3.99 ± 0.03 d	05.94 ± 0.40 c
AMV	1.97 ± 0.01 e	2.46 ± 0.04 c	401.1 ± 8.74 a	9.12 ± 0.43 b	03.82 ± 0.11 d
CuO-NPs	4.01 ± 0.05 c	3.33 ± 0.06 ab	388.9 ± 1.68 a	5.92 ± 0.14 c	07.13 ± 0.55 c
Curative treatment	5.43 ± 0.17 a	3.72 ± 0.29 a	389.1 ± 3.24 a	9.79 ± 0.28 a	16.93 ± 1.11 a
Protective treatment	3.21 ± 0.05 d	3.01 ± 0.05 b	393.8 ± 5.16 a	9.22 ± 0.09 ab	10.59 ± 1.07 b

Differences between groups were determined using a one-way analysis of variance (ANOVA) and Tukey’s HSD test at the *p* ≤ 0.05 significance level in the CoStat statistical package. Statistical significance was indicated alphabetically above the histogram in ascending order, whereas a > b > c > d

### Impact of CuO-NPs on phenolic and flavonoid content

The result of phenolic content analysis (Table 4) revealed that AMV treatment record the highest value (401.1 ± 8.74) while the CuO-NPs showed the lowest value of 388.9 ± 1.68. among five treatments, there are no significant differences were observed between the treatments. This contrasts with a research indication of the influence of CuO-NPs on phenol production [58], which has the highest total phenolic content observed in roots treated with CuO-NPs. Phenols enter resistance to disease in several ways, like cell wall lignification and hypersensitive cell death. Accordingly, the increase in phenolics gives higher protection against pathogens [73]. For flavonoid, the highest phenolic content (9.79 ± 0.28) showed in curative treatment followed protective and AMV treatments with 9.22 ± 0.09 and 9.12 ± 0.43, respectively (Table 4). The control plant reported the lowest level of flavonoid content (3.99 mg/g). Consequently, the application of CuO-NPs resulted in elevation of plant flavonoid content. It has been reported that the foliar spray of CuO-NPs positively affected the production of phytochemical compounds such as total flavonoids, chlorophyll, and antioxidant capacity [74, 75]. The obtained results showed that leaves infected with AMV had elevated levels of both total phenolic and flavonoid contents. Hence, the elevation of overall phenolic and flavonoid levels in leaves affected by a virus likely plays a role in enhancing the immune response against viral infection. The results are similar to those of Lan et al. [76], who found that CMV-infected *Passiflora edulis* leaves had higher amounts of polyphenols and flavonoids by 26.1% and 48.1%, respectively.

### Impact of CuO-NPs on antioxidant enzymatic activities

Plants possess a plentiful supply of both enzymatic and non-enzymatic antioxidants, enabling them to effectively remove excessive reactive oxygen species (ROS) and play a vital role in regulating ROS homeostasis. CAT, POX, and SOD are crucial enzymes in antioxidant activity and enhance the primary defense mechanism against cellular oxidative damage [77]. Among four different antioxidant enzyme activities (Table 5), SOD levels were much higher in the CuO-NPs and curative treatments (29.02 ± 0.54 and 27.99 ± 0.65 µM/g f.wt., respectively) compared to the control (7.60 ± 0.48). The AMV treatment reported 22.02 ± 0.57 µM/g f.wt., while the protective treatment exhibited a considerable increase with 23.71 ± 0.31 µM/g f.wt. Regarding PPO, no significant variations were found across the treatments. The AMV treatment exhibited the maximum value (7.49 ± 0.15), while the CuO-NPs treatment showed the lowest value (6.48 ± 0.60). Likewise, the AMV, curative, and protective treatments showed the highest level of POX with 3.12 ± 0.52, 2.83 ± 0.18, and 2.71 ± 0.52 µM/g f.wt., respectively, compared to the control that exhibited the lowest level with 1.63 ± 0.36 µM/g f.wt. For CAT, the AMV treatment (137.6 ± 4.59) exhibited a significant change compared to the protective treatment (129.2 ± 1.49), while no significant change was reported between the other three treatments. The CuO-NPs, curative, and control treatments exhibited 134.6 ± 0.09, 132.6 ± 1.68, and 131.1 ± 2.09 µM/g f.wt., respectively. Accordingly, the obtained results were in agreement with previous research that showed a significant increase of antioxidant enzymes (SOD and CAT) upon treatment of *Oryza sativa* and *Brassica rapa* with CuO-NPs [17, 78, 79]. It was reported that the viral infection is associated with increasing antioxidant enzyme activity [50]. Treated tomato plants with Si-NPs exhibited elevated POX and PPO activity, indicating enhanced systemic resistance to tomato yellow leaf curl virus (TYLCV). Furthermore, the enhanced systemic resistance correlated with a reduction in the severity of the TYLCV, as it delayed the appearance of viral symptoms in the treated tomato plants compared to the untreated control plants [80]. The enhancement of POX, PPO, and TSP activities in tomatoes post-infection with TMV and PVY resulted in a notable increase in photosynthetic pigments, accompanied by an elevation in total soluble phenols and free proline levels [66].

**Table 5:** Impact of CuO-NPs_100ppm_ on the enzymatic antioxidant activities and oxidative stress markers of tobacco plants infected with AMV (28 days after inoculation). Healthy tobacco plants (control); tobacco plants inoculated with virus only (AMV); tobacco plants treated with CuO-NP100ppm alone (CuO-NPs); tobacco plants inoculated with CuO-NPs_100ppm_ 48 h after AMV inoculation (curative treatment); tobacco plants inoculated with CuO-NPs_100ppm_ 48 h before AMV inoculation (protective treatment)

Treatments	Enzymatic Antioxidant activities ± SD				Oxidative Stress Markers ± SD	
	SOD	PPO	POX	CAT	MDA	H_2_O_2_
Control	07.60 ± 0.48 d	6.79 ± 0.54 a	1.63 ± 0.36 b	131.1 ± 2.09 ab	093.9 ± 3.78 c	06.17 ± 0.68 c
AMV	22.02 ± 0.57 c	7.49 ± 0.15 a	3.12 ± 0.52 a	137.6 ± 4.59 a	326.9 ± 9.72 a	11.99 ± 0.71 a
CuO-NPs	29.02 ± 0.54 a	6.48 ± 0.60 a	2.41 ± 0.26 ab	134.6 ± 0.09 ab	097.4 ± 2.37 bc	07.41 ± 0.38 b
Curative treatment	27.99 ± 0.65 a	6.90 ± 0.45 a	2.83 ± 0.18 a	132.6 ± 1.68 ab	117.7 ± 10.09 b	07.02 ± 0.65 b
Protective treatment	23.71 ± 0.31 b	6.89 ± 0.46 a	2.71 ± 0.52 a	129.2 ± 1.49 b	103.9 ± 11.16 bc	07.16 ± 0.51 b

Differences between groups were determined using a one-way analysis of variance (ANOVA) and Tukey’s HSD test at the *p* ≤ 0.05 significance level in the CoStat statistical package. Statistical significance was indicated alphabetically above the histogram in ascending order, whereas a > b > c > d

### Impact of CuO-NPs on oxidative stress markers

In our study, we measured two markers of oxidative stress, MDA and H_2_O_2_ (Table 5). The results indicate that MDA had the highest level in the AMV treatment (326.9 ± 9.72 mg/g). There were no significant differences observed between the three curative, protective, and CuO-NPs treatments, while the curative treatment (117.7 ± 10.09) significantly changed from the control treatment (93.9 ± 3.78). Similarly, the AMV recorded the highest value of H_2_O_2_ (11.99 ± 0.71 mg/g), followed by CuO-NPs and protective and curative treatments with 7.41 ± 0.38, 7.16 ± 0.51, and 7.02 ± 0.65, respectively. The control plant showed the lowest level of H_2_O_2_ (6.17 ± 0.68). It is known that Cu-NPs generate oxidative stress, cause the disassembly of viruses or bacterial membranes, and can interfere with virus activity [81]. These agreements with Omar et al. [82] who indicated the speedy development of H_2_O_2_ in *Ch. amaranticolor* leaves when infected by TMV compared to the control plant. Multiple reports have assessed that the viral infection is linked to an increase in MDA and H_2_O_2_ levels [69, 82]. The research by Bhatnagar et al. [58] consistently demonstrated that CuO-NPs increased the production of ROS and H_2_O_2_. Similarly, Upadhyay and Panda [83] reported that CuO-NPs resulted in an increased synthesis of H_2_O_2_ and superoxide radicals in leaf cells and roots. The small rise in ROS production, (MDA and H_2_O_2_), after CuO-NPs were applied to plant leaves suggests that nanoparticles may not be harmful to plant tissues [15, 16, 18].

### Impact of CuO-NPs on expression levels of defense-related genes

**Fig. 5:**
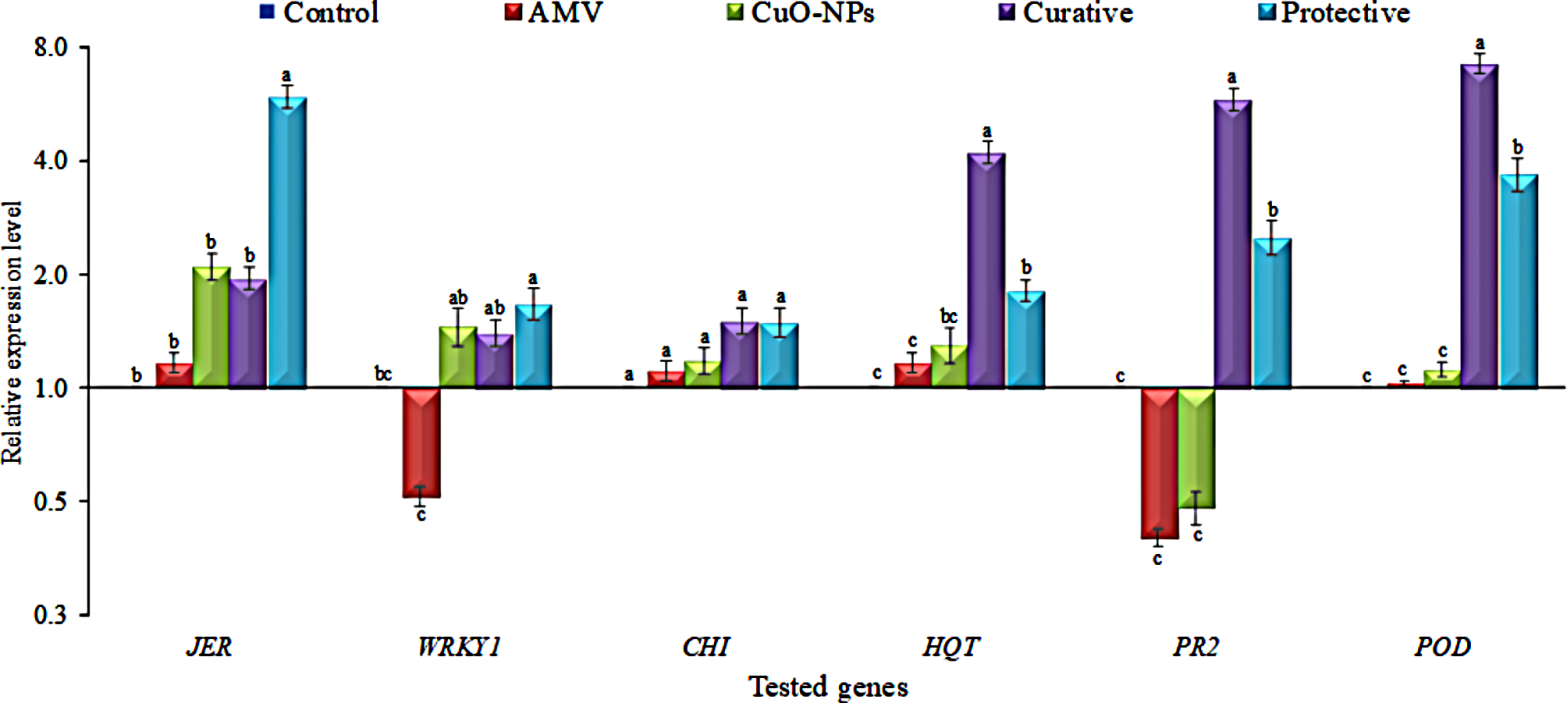
Impact of the foliar application of CuO-NPs synthesis using *H. salicornicum* extract on the jasmonic acid pathway (JERF3 and WRKY1), polyphenolic pathway (CHI and HQT), SA-signaling pathway (PR2 and POD) genes on tobacco plants under greenhouse conditions. Control: tobacco plants; AMV: tobacco plants inoculated with AMV; CuO-NPs100ppm: tobacco plants treated with CuO NPs100ppm; AMV + CuO-NP100ppm: tobacco plants inoculated with CuO-NPs100ppm 48 h after AMV inoculation (curative); CuO-NP100ppm + AMV: tobacco plants inoculated with CuO-NPs100ppm 48 h before AMV inoculation (protective)

The RT-qPCR results (Fig. 5) indicated that there was a notable differentiation across treatments in the expression of the two genes involved in the Jasmonic biosynthesis pathway, namely (*JERF3* and *WRKY1*). JA plays a vital role in plant antiviral defense [84]. For *JERF3*, the protective treatment showed the highest relative expression level, which was 5.95 times greater than the control. However, no significant differences were detected between the other treatments and the control group. Similarly, the *WRKY1* gene showed the highest transcriptional level in the protective treatment, with a transcriptional level 1.67-fold higher than the control. The CuO-NPs and curative treatment significantly increased the relative expression level of *WRKY1* with a 1.46- and 1.41-fold change compared to the control. Landa et al. [85] observed increased levels of SA and JA in *Arabidopsis thaliana* as well as the transcription of genes involved in defensive signaling. upon exposure to CuO-NPs. Also, in *N. benthamiana*, Cai et al. [86] found increased levels of SA but no change in the JA level was noted. Therefore, the previous results imply that the particular interaction between NPs and plants, application period, and dose determine the development of a specific plant hormone. On the other hand, the AMV treatment showed downregulation, with a relative expression level of 0.51 lower than the control. Additionally, a number of studies have shown that in plants infected with tomato yellow leaf curl Sardinia virus, RRSV, and RBSDV, the expression of genes involved in JA production is decreased [87, 88]. This demonstrates that viruses have the ability to boost viral infection by targeting JA biosynthesis. Liu et al. [89] proposed that the utilization of CuO-NPs can inhibit TMV infection without inducing noticeable phytotoxicity by directly modifying the TMV structure and activating plant immune responses via alterations in the JA, SA, and ET signaling pathways.

Furthermore, our investigation revealed the impact of two polyphenolic biosynthesis pathway genes (*CHI* and *HQT*). Chalcone isomerase (*CHI*) is an enzyme that facilitates the conversion of naringenin chalcone to naringenin, which is essential for the generation of flavonoids in various plant tissues. Hydroxycinnamoyl CoA quinate transferase (*HQT*) is an enzyme found in solanaceous plant species that plays a key role in the main pathway for chlorogenic acid synthesis. In the current study, the transcriptional level of the *CHI* gene indicated no differences between the treatments, where the highest expression level (1.51-fold) was observed in the curative treatment and the lowest was shown in the AMV treatment (1.11-fold) compared to the control. For *HQT*, the curative treatment significantly induced the transcriptional level of *HQT* by 4.23-fold, followed by the protective treatment by 1.82-fold higher than the control. According to Raigond et al. [90], ZnO-NPs also increased the anthocyanin and total phenolic content in potato plants. On the other hand, the AMV treatment exhibited an expression level of 1.17-fold with no significant change from the control.

PR proteins are a crucial element of a plant’s immune system since they function as diagnostic molecular markers of plant defense signaling pathways [91]. According to Ali et al. [92], the overexpression of the SA signaling pathway is indicated by the activation of the *PR1*, *PR2*, and *PR5* genes. The application of SiO2-NPs and ZnO-NPs caused the uninfected tobacco plants to upregulate the SA-inducible PR genes, *PR1* and *PR2*, and Fe3O4-NPs had a similar impact [86]. Our results showed that SA-signaling pathway genes (*PR2* and *POD)* reported the highest transcriptional level in curative and protective treatments. For *PR2*, the curative and protective treatments resulted in a considerable increase in transcriptional levels, with a 5.83-fold and 2.52-fold greater expression, respectively, compared to the control. The transcriptional levels of CuO-NPs and AMV treatments fell by 0.48-fold and 0.41-fold, respectively. However, no significant difference was seen compared to the control. In the same manner, the *POD* transcript profile exhibited significant increases in the curative and protective treatments, with a 7.28-fold and 3.69-fold higher expression, respectively, compared to the control. This was compatible with Si-NPs-treated tomato plants showed higher POD activities, indicating improved systemic resistance against TYLCV [80]. Under greenhouse conditions, ZnO-NPs were also found to improve resistance against CMV in brinjal plants [93]. However, no substantial differences were seen between the AMV and CuO-NPs treatments and the control. POD plays a crucial role in preventing severe damage caused by ROS resulting from viral infections and causing harm to plant cells. POD can eliminate H_2_O_2_, reduce the presence of free radicals, and also safeguard the cytoplasmic membrane [94, 95].

### Molecular docking interaction between CuO-NPs and AMV genes

**Fig. 6:**
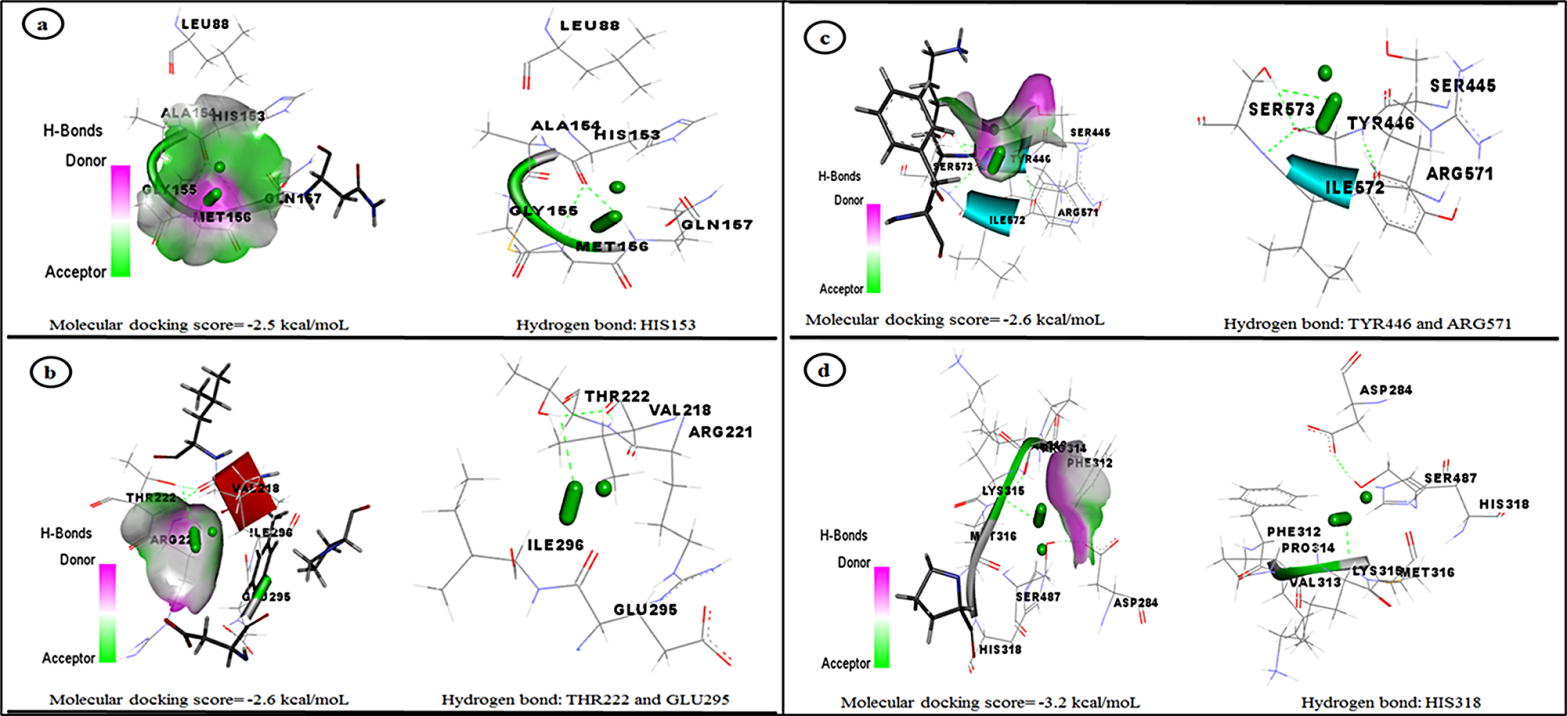
Molecular docking interaction of CuO-NPs synthesis using *H. salicornicum* extract and Alfalfa mosaic virus’s genes. **a**: capsid protein; **b**: movement protein; **c**: RNA-directed RNA polymerase 2a; **d**: replication protein 1a

Until now, the exact mechanism by which green-synthesized CuO-NPs exhibit antiviral properties remains unclear; however, their effectiveness against various viruses is probably attributed to the interaction of CuO-NPs with viral proteins, which may hinder viral entry into host cells. CuO-NPs may exhibit antiviral properties via interacting with the viral genome or inhibiting processes essential for viral replication [96]. The present investigation involves a docking study that anticipates interaction patterns between AMV proteins and CuO-NPs, potentially elucidating their antiviral properties. Figure 6 depicts the investigation of the molecular docking contact and scores between CuO-NPs, which were generated using *Haloxylon salicornicum* extract, and the target proteins of the AMV (capsid protein, movement protein, RNA-directed RNA polymerase 2a, and replication protein 1a). Figure 6a illustrates the strong attraction (affinity) between CuO and the HIS153 residue through a hydrogen bond. This interaction occurs within the binding site of the capsid protein and is characterized by an energy of -2.5 kcal/mol. Besides that, CuO made hydrogen bonds with the THR222 and GLU295 parts in the movement protein’s binding site. The energy value associated with this interaction was − 2.6 kcal/mol, as shown in Fig. 6b. There was a specific interaction between the CuO and the TYR446 and ARG571 residues that were in the binding region of RNA-directed RNA polymerase 2a. This interaction resulted in the formation of hydrogen bonds and an energy value of -2.6 kcal/mol, as depicted in Fig. 6c. Also, CuO made a hydrogen bond with the HIS319 residue at the replication protein 1a binding site. This bond had an energy of -3.2 kcal/mol (Fig. 6d). The results showed that CuO can inhibit four proteins that are important for the replication and spreading of the AMV. This inhibition hinders the viral replication and invasion process, as these proteins are crucial for the virus’s replication and spreading [97, 98]. Overall, the efficiency of green-produced CuO-NPs provides good prospects for the discovery of new antiviral agents targeting plant viruses, and further investigation of the mechanism of action is required for the development of more effective approaches for viral diseases management.

## Conclusions

The application of *H. salicornicum* aqueous extract in the biosynthesis of CuO nanoparticles yielded spherical and hexagonal structures measuring between 20 and 70 nm, alongside a diverse array of functional groups originating from plant secondary metabolites. Under greenhouse conditions, the foliar application of CuO-NPs at 100 µg/L showed a positive impact on different growth parameters and physicochemical properties of tobacco plants. In addition, when compared to virally infected plants that were not CuO-NPs-treated, the plants showed a significant drop in oxidative stress indicators. Notably, the impact on AMV accumulation levels resulted in a reduction of approximately 97% for both curative and protective treatments, with a preference for curative treatments that did not exhibit any viral symptoms. There were also significant differences in the levels of total chlorophyll, phenolic, flavonoid, and content, as well as other antioxidant enzymes such as SOD, PPO, POX, and CAT. Furthermore, there was an increase in the expression levels of certain genes in the polyphenolic pathway, the SA-signaling pathway, and the jasmonic pathway. The molecular docking analysis demonstrated that CuO-NPs exhibits inhibitory effects on four essential proteins that play a role in the replication and spread of the AMV. Consequently, CuO-NPs may serve as a sustainable foliar antiviral solution, and we suggest integrating both curative and protective measures to prevent AMV infection across various susceptible hosts. However, additional examination of the effectiveness of this method against different plant viruses is necessary before its broad implementation across diverse crops and viruses, along with a focus on the feasibility of CuO-NPs for extensive open-field application. Finally, the long-term impacts of CuO-NPs on soil ecosystems and non-target organisms remain uncertain. So, more research is needed to fully understand what effects CuO-NPs might have when they are present with abiotic stressors or other contaminants in the environment. Moreover, the genotoxicity of CuO-NPs is little examined, signifying a necessity for additional research in this area going forward.

## Data Availability

The data that supports the findings is included in the publication or is available from the corresponding author upon reasonable request.
